# The exon junction complex core factor eIF4A3 is a key regulator of HPV16 gene expression

**DOI:** 10.1042/BSR20203488

**Published:** 2021-04-07

**Authors:** Koceila Meznad, Philippe Paget-Bailly, Elise Jacquin, Anne Peigney, François Aubin, Michaël Guittaut, Christiane Mougin, Jean-Luc Prétet, Aurélie Baguet

**Affiliations:** 1 Université Bourgogne Franche Comté, France; 2 EA3181, UFR Santé, F-25000, Besançon, France; 3 INSERM, UMR1098, Interactions Hôte-Greffon-Tumeur/Ingénierie Cellulaire et Génique, F-25000, Besançon, France; 4 INSERM, U1231, Université Bourgogne Franche Comté, Dijon, France; 5 Centre Hospitalier Régional Universitaire, 3 Bvd Alexandre Fleming, Besançon, France; 6 DimaCell Platform, Université Bourgogne Franche-Comté, F-25000 Besançon, France; 7 Centre National de Référence Papillomavirus, F-25000 Besançon, France

**Keywords:** Cervical cancer, DDX48/eIF4A3, Exon Junction Complex, HPV

## Abstract

High-risk human papillomavirus (hrHPVs), particularly HPV16 and HPV18, are the etiologic factors of ano-genital cancers and some head and neck squamous cell carcinomas (HNSCCs). Viral E6 and E7 oncoproteins, controlled at both transcriptional and post-transcriptional levels, drive hrHPVs-induced carcinogenesis. In the present study, we investigated the implication of the DEAD-box helicase eukaryotic translation initiation factor 4A3 (eIF4A3,) an Exon Junction Complex factor, in the regulation of HPV16 gene expression. Our data revealed that the depletion of the factor eIF4A3 up-regulated E7 oncoprotein levels. We also showed that the inhibition of the nonsense-mediated RNA decay (NMD) pathway, resulted in the up-regulation of E7 at both RNA and protein levels. We therefore proposed that HPV16 transcripts might present different susceptibilities to NMD and that this pathway could play a key role in the levels of expression of these viral oncoproteins during the development of HPV-related cancers.

## Introduction

Human papillomaviruses (HPVs) are small non-enveloped viruses with circular double-stranded DNA genomes. More than 200 types of HPVs infect humans [1] and are classified according to their carcinogenic potential. High-risk HPV types (hrHPVs) (including HPV16 and HPV18) are the etiological agents of cervical and anal carcinomas and frequently found in head and neck squamous cell carcinomas (HNSCCs), and low-risk HPV types (lrHPVs) (including HPV6 and HPV11) are associated with benign lesions such as genital warts [2,3]. In most cases, HPV is cleared by the immune system in the first year after infection. However, persistence of hrHPV infection is associated with an increased risk of developing high-grade squamous intra-epithelial lesions (HSIL), corresponding to the histological grade of Cervical Intraepithelial Neoplasia 2/3 (CIN). In this case, HPV infection is most often abortive and associated with a deregulation of viral gene expression and a high risk of progression to cervical cancer [4–7].

The HPV16 genome comprises a long control region (LCR) and eight open reading frames (ORFs) encoding early (E1, E2, E4, E5, E6 and E7) and late (L1 and L2) viral proteins. The early promoter p97 located in the LCR drives the expression of genes coding early proteins whereas late proteins are produced from transcripts regulated by the late promoter p670. hrHPV-driven carcinogenesis is most often related to viral genome integration into the host genome, leading to E6 and E7 oncoproteins overexpression [5,8–10]. Both HPV16 E6 and E7 proteins present pleiotropic effects by interacting with multiple host cell proteins, such as p53 and pRb tumor suppressors, respectively [11–14]. Continuous expression of HPV16 E6 and E7 proteins has been demonstrated to be essential for the initiation and the maintenance of cancer [15].

In addition to transcriptional regulation, HPV16 RNA processing largely contributes to the regulation of viral protein expression. For instance, HPVs take advantage of alternative splicing regulated by cellular splicing factors to allow for the production of multiple viral protein-encoding mRNAs [16,17]. Indeed, p97-derived HPV16 transcripts are submitted to alternative splicing which generates more than a dozen different polycistronic mRNAs encoding early proteins [16,18]. At least four cryptic splicing sites have been identified in HPV16 E6 and E7 ORFs: one splicing donor site, SD226, and three splicing acceptor sites, SA409, SA526 and SA742, leading to the putative expression of truncated proteins, respectively named E6*I, E6*II and E6⁁E7 [19–21].

The eukaryotic translation initiation factor 4A3 (eIF4A3, also known as DDX48) is a DEAD-box RNA helicase implicated in the formation of the Exon Junction Complex (EJC), unlike its homologous eIF4AI and eIF4AII which are required for translation initiation. The EJC is a multiprotein complex assembled on to the pre-messenger RNA (pre-mRNA) during splicing. The complex is recruited 20–24 nucleotides upstream of the exon–exon junction, in a sequence-independent manner [22]. The EJC core is composed of four proteins, named eIF4A3, Y14 (RBM8A), MAGOH and MLN51 (CASC3), which acts as a binding platform to recruit other peripheral EJC factors [23–27]. Consequently, the composition of the EJC evolves all along the nuclear and cytoplasmic mRNA life [28,29]. Ultimately, EJC factors are removed by the translation machinery during the first round of translation [30,31]. The EJC greatly influences mRNA fate by affecting RNA processing: *(i)* the EJC regulates the splicing process [25,32–34] and mRNA transport [35,36], *(ii)* it promotes nuclear export [37–39] and translation [40–42] and *(iii)* it plays a crucial role in the mRNA surveillance pathway [43]. Indeed, it has been shown that the EJC allows for the recognition of Premature Termination Codons (PTCs) on transcripts and their targeting for degradation by the nonsense-mediated mRNA decay (NMD). So, even if it is clear that EJC factors are key players in post-transcriptional regulation of gene expression, the role of eIF4A3 in HPV RNA processing has never been studied. In the present study, we therefore investigated, for the first time, the implication of eIF4A3 in the regulation of HPV16 E6 and E7 oncoprotein expression.

## Materials and methods

### Overall survival analysis based on The Cancer Genome Atlas data

Correlation analysis between eIF4A3 expression and overall survival of patients presenting cervical cancer were based on The Cancer Genome Atlas (TCGA) Research Network (http://www.cancer.gov/tcga). The graph and log-rank analysis were generated from the Human Protein Atlas (http://www.protein-atlas.org) [44]. The 291 cervical cancer patients were stratified by the median of eIF4A3 mRNA expression.

### Cell culture

Human cervical cancer-derived cell lines, C-33 A (HPV-negative, ATCC HTB-31), SiHa (HPV16-positive, ATCC® HTB-35) and Ca Ski (HPV16-positive, ATCC® CRL-1550) were obtained from the American Type Culture Collection. C-33 A and SiHa cells were cultured in DMEM and Ca Ski in RPMI-1640 medium (Lonza, Verviers, Belgium) supplemented with 10% fetal bovine serum (Eurobio, Courtaboeuf, France). Cells were incubated at 37°C in a humidified atmosphere with 5% CO_2_. All cell lines were routinely tested for mycoplasma contamination.

### siRNAs, expression vectors and chemical reagents

Negative control (SR-CL000-005) and eIF4A3 (target sequence: 5′-AGACATGACTAAAGTGGAA-3′) targeting siRNA duplexes were purchased from Eurogentec (Liege, Belgium). The peGFP-eIF4A3, peGFP-MLN51 and peGFP-Dcp1a vectors were a kind gift from Dr Catherine Tomasetto [45] and Dr Fabienne Mauxion [46] (IGBMC, Illkirch, France). The translation inhibitor cycloheximide (CHX) was purchased from Sigma and used at the concentration of 100 μg/ml. The nucleotide analogs 5-aza-2′-deoxycytidine (5-azadC) and 5-azacytidine (5-azaC) were purchased from Epigentek and StressMarq Biosciences, respectively, and used at a concentration range from 0.5 to 10 μM.

### Antibodies

Mouse antibodies anti-E7 (1/1000), anti-E6 2E-3F8 (1/1000) and anti-βactin AC-15 (1/10000) were purchased from Santa Cruz, Euromedex and Sigma, respectively. Mouse and rabbit anti-eIF4A3 antibodies were generously provided by Catherine Tomasetto (I.G.B.M.C., Illkirch, Strasbourg). HRP-conjugated goat anti-mouse and anti-rabbit secondary antibodies were purchased from BD Pharmingen. Alexa 488-labeled goat anti-rabbit and Cy3-labeled donkey anti-mouse secondary antibodies used for immunofluorescence were purchased from Jackson ImmunoResearch.

### Transfection

Cells were plated in six-well plates 1 day before transfection so that they were 60–75% confluent on the day of the transfection. siRNA transfections were performed using the Lipofectamine 2000 reagent (Invitrogen, Carlsbad, U.S.A.) following the supplier’s recommendations. Cells were then cultured for 48 h in complete medium before harvesting. Plasmid transfections were performed using the JetPEI reagent (Polyplus-transfection, Illkirch, France) according to the manufacturer’s instructions. Cells were incubated 24 h in complete medium before harvesting.

### RNA-FISH

Cells were cultured on coverslips in 24-well plates. Plasmid or siRNA transfections were performed as described above. After transfection, cells were fixed in 4% paraformaldehyde for 30 min at room temperature. RNA-FISH assays were performed using the RNAscope® Fluorescent Multiplex Assay (Advanced Cell Diagnostics Bio, Milan, Italy) following the supplier’s recommendations. Briefly, cells were dehydrated with increasing concentrations of EtOH (50, 70 and 100% for 2 min) and rehydrated with decreasing concentrations of EtOH (70 and 100% for 2 min). Cells were then incubated with Protease III for 10 min at room temperature and subjected to RNAscope probe hybridization and counterstaining with DAPI. Slides were mounted in Vectashield (Polysciences Inc.) and subjected to confocal microscopy (Zeiss LSM 800 AiryScan).

### RT-PCR and RT-qPCR

Total RNAs were extracted using the Ribozol reagent (Amresco) and 500 ng total RNAs were retro-transcribed into cDNA using the Maxima first-strand cDNA synthesis kit (ThermoScientific) according to the manufacturer’s instructions. For PCR, cDNAs were amplified using the DreamTaq polymerase (Thermo Scientific) according to the manufacturer’s instructions. PCR products were then analyzed on a 1 or 2% (w/v) agarose gel prepared in 1× Tris-Borate-EDTA buffer. Quantitative PCR was performed using the Power SYBR Green PCR mix (Life Technologies) according to the manufacturer’s instructions and analyzed in a StepOnePlus Real-Time PCR system (Applied Biosystems). Primer sequences are listed in the Supplementary Table S1. 18S rRNA or β2-microglobulin expression levels were used as internal normalization standards [47]. The 2^−ΔΔ*C*_t_^ method was used for relative mRNA quantification. Unilateral Student’s *t* tests were used to analyze statistical significance of RT-qPCR experiments (*: *P*<0.05; **: *P*<0.01; ***: *P*<0.001).

### Protein extraction and Western blotting

Cell lysis was performed using RIPA buffer (50 mM Tris-HCl pH7.4, 150 mM NaCl, 1% NP40, 0.5% Na deoxycholate, 1 mM EDTA) supplemented with 30 μg/ml of anti-protease mixture (Roche Diagnostics, Mannheim, Germany) followed by 5-s sonication. Following centrifugation at 10000×***g*** for 10 min at 4°C, protein concentrations were determined using the Bio-Rad Protein Assay (Bio-Rad, Munich, Germany) according to the manufacturer’s instructions. Proteins were then separated by SDS/PAGE and transferred on to Amersham™ Hybond™-PVDF membranes (GE Healthcare, Munich, Germany). Membranes were blocked overnight at 4°C with 5% nonfat milk, probed during 1 h with primary antibodies (see above) and then with HRP-conjugated secondary antibodies. Chemiluminescent signals were detected using the PierceECL 2 Western Blotting Substrate (Thermo Scientific). The signals were then analyzed and quantified using the ChemiDoc XRS+ system (Bio-Rad), and the Image Lab software (5.1, Bio-Rad).

### RNA-immunoprecipitation

Cells were lysed in NET-2 buffer (50 mM Tris-HCl pH7.4, 150 mM NaCl, 10 mM MgOAc, 0.05% (v/v) NP-40, 1 mM DTT, protease inhibitor (Roche Diagnostics), 80 U/ml RNAse OUT (Invitrogen)), sonicated five times for 1 s, and centrifuged at 16100×***g*** for 10 min at 4°C. Then, the samples were washed with NET-2 buffer/300 mM NaCl followed by a centrifugation at 500×***g*** for 5 min at 4°C. Then, NET-2 buffer/300 mM NaCl and 1 mg/ml heparin was added and incubated under agitation for 10 min at 4°C, followed by a centrifugation step at 500×***g*** for 5 min at 4°C. After eight washing steps using NET-2 buffer and a centrifugation step at 500×***g*** for 5 min at 4°C, protein and antibodies’ complexes were eluted in 50 μl Elution buffer (0.1 M Tris-HCl pH 6.8, 20% (v/v) glycerol, 4% (w/v) SDS, 12% (v/v) βMercapto-EtOH) followed by a centrifugation step at 500×***g*** for 5 min at 4°C. Eluates were finally split into two fractions: one fraction was used to perform Western blotting and the other was subjected to RNA extraction followed by RT-qPCR analysis.

## Results

### High levels of eIF4A3 is associated with a good prognosis in cervical cancers

**Figure 1:**
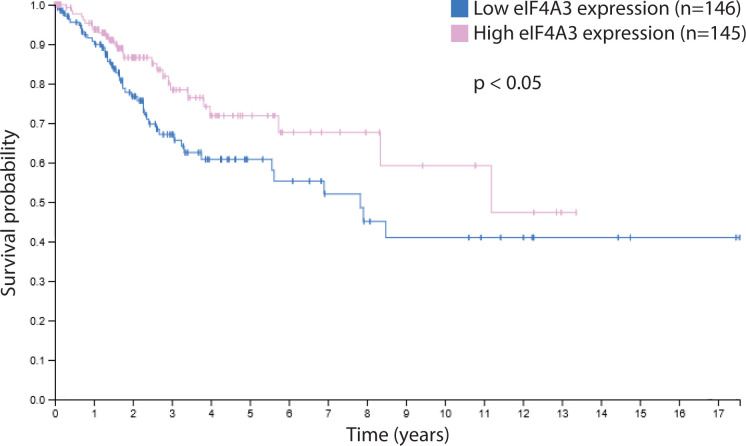
eIF4A3 expression in cervical precancerous and cancerous lesions Kaplan–Meier overall survival of the 291 TCGA cervical cancer samples (CESC) stratified according to the median of eIF4A3 mRNA expression.

First, we evaluated the prognostic value of eIF4A3 expression in cervical cancers using TCGA database. Among the 291 samples available, the 5-year survival of patients presenting high levels of eIF4A3 (above median of eIF4A3 level) was 76%, compared with 59% for patients presenting low levels of eIF4A3 (below median of eIF4A3 level) (*P*<0.05) (Figure 1). These data demonstrated that a high expression of eIF4A3 was an indicator of good prognosis for cervical cancer patients.

### The expression of the HPV16 E7 oncoprotein was increased in eIF4A3-depleted cells

**Figure 2:**
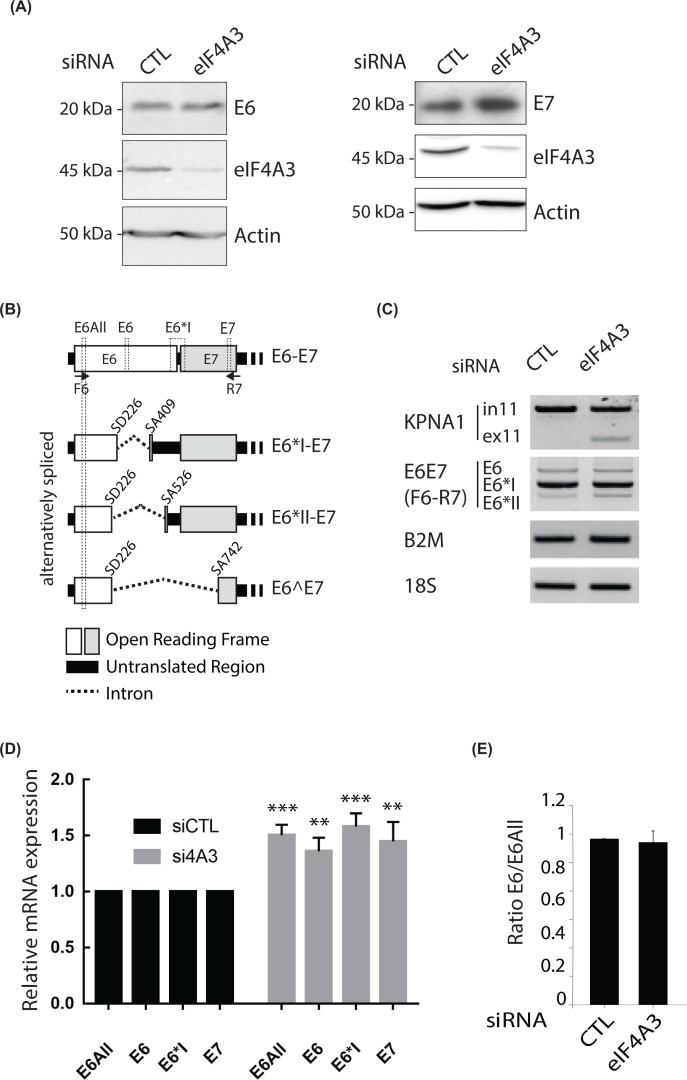
The depletion of eIF4A3 increases HPV16 E7 protein expression (**A**) Western blotting analyses showing endogenous E6 (left panel), E7 (right panel) and eIF4A3 protein levels in CTL or eIF4A3-depleted SiHa cells. β*-*actin was used as a loading control. (**B**) Representative scheme of unspliced and alternatively spliced HPV16 early transcripts. Real-time PCR primer sets are in dotted lines (E6All, E6, E6*I and E7) and RT-PCR primer sets are represented by arrows (F6 and R7). (**C**) RT-PCR showing alternative splicing patterns of HPV16 E6/E7 transcripts in CTL or eIF4A3-depleted SiHa cells. KPNA1 was used as a positive control of eIF4A3 depletion efficiency. B2M and 18S were both used as loading controls. (**D**) RT-qPCR analysis of HPV16 E6all, E6, E6*I and E7 expression in SiHa cells treated with siRNA-CTL or siRNA-eIF4A3 for 48 h. 18S was used for normalization. Data are represented as means of seven independent experiments. Error bars = s.e.m.; **P<0.01; ***P<0.001; two-tailed Student’s *t* test. (**E**) E6/E6All ratio of mRNA levels quantified by real-time PCR in CTL or eIF4A3-depleted SiHa cells. Data are represented as means of seven independent experiments. Error bars = s.e.m.

To determine whether eIF4A3 could regulate HPV16 gene expression, we performed siRNA-mediated depletion of eIF4A3 in the HPV16-positive cervical carcinoma SiHa cell line. We observed that the depletion of eIF4A3 had no effect on E6 protein levels (Figure 2A, **left panel**). Interestingly, the depletion of eIF4A3 led to an increase in E7 protein levels in eIF4A3-depleted cells compared with control samples (Figure 2A, **right panel**). Several alternatively spliced transcripts produced E6 and E7 oncoproteins following the use of one donor splice site (SD226) and three acceptor splice sites (SA409, SA526 and SA742) (Figure 2B). The recognition by the spliceosome of the combinations of SD226/SA409 or SD226/SA526 splicing sites produced two different spliced transcripts encoding the E7 protein and potentially two truncated E6 proteins, E6*I and E6*II, respectively. A fusion protein called E6⁁E7 is also potentially encoded by the spliced transcript SD226/SA742. Finally, the full-length E6 protein could exclusively be encoded by the unspliced transcript. In SiHa cell line, spliced transcripts potentially encoding both E7 and E6-truncated proteins were mainly expressed (Supplementary Figure S1). Next, we asked whether the deregulation of E7 expression mediated by eIF4A3 could lead to a modulation of the alternative splicing pattern of E6/E7 transcripts. To answer this question, we used specific primer sets developed for RT-PCR (F6 and R7 primers) or RT-qPCR (E6All, E6, E6*I and E7 primers) to simultaneously, or individually, detect HPV16 E6/E7 alternative transcripts (see Figure 2B). The representative RT-PCR results, obtained using the primers (F6-R7) targeting early HPV16 transcripts, are presented in Figure 2C. The KPNA1 transcript was used as a positive control since it has been previously reported that the depletion of eIF4A3 led to an increase in exon skipping of the constitutive exon 11 of KPNA1 [34]. As expected, the eIF4A3 knockdown indeed led to an increase in the exclusion of the exon 11 of KPNA1. However, the depletion of eIF4A3 did not alter the splicing profile of HPV16 early transcripts. Also, we performed RT-qPCR using E6All, E6, E6*I and E7 primers and observed a low but statistically significant elevation of HPV16 early transcripts (1.6-fold increase) in eIF4A3-depleted cells (Figure 2D). Finally, both E6/E6All ratios calculated in siRNA-CTL and siRNA-eIF4A3 treated cells were ∼1, confirming that the depletion of eIF4A3 did not affect the splicing pattern of HPV16 early transcripts (Figure 2E). Altogether, these data indicated that eIF4A3 induced an increase in E7 oncoprotein levels and that this increase was not linked to a differential alternative splicing.

### HPV16 RNA localization in eIF4A3-depleted cells

**Figure 3:**
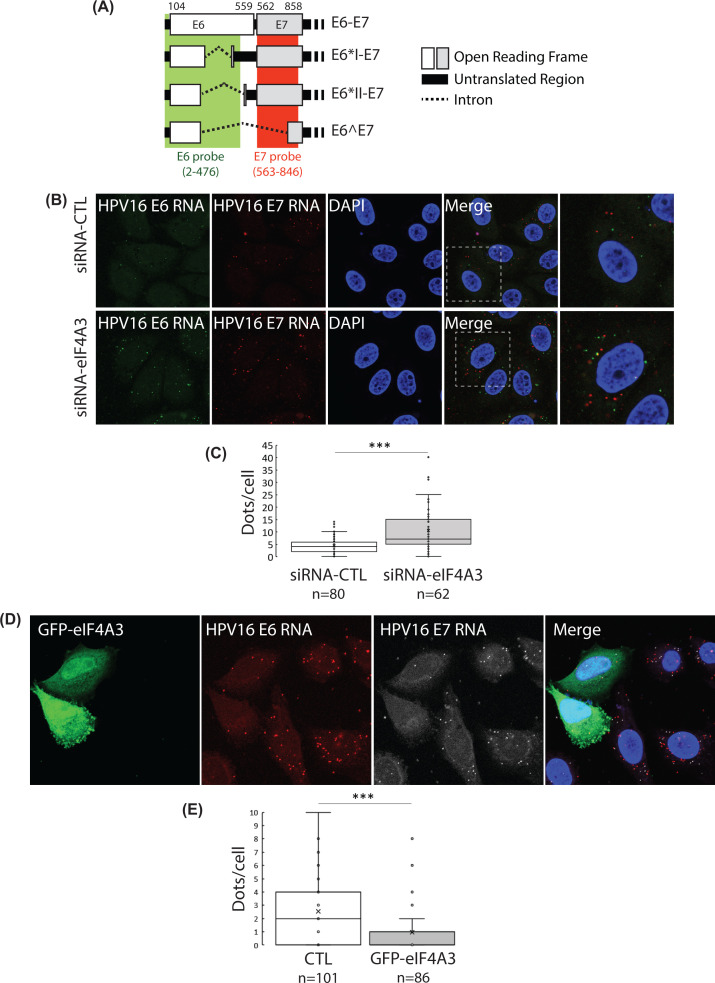
Subcellular localization of HPV16 E6/E7 mRNA using RNAscope (**A**) Representative scheme of E6 probe (green) and E7 probe (red) recognizing HPV16 transcripts. (**B**) Transcripts were detected as punctate dots in eiF4A3-depleted SiHa cells or control cells by confocal microscopy (Z-section). Nuclei were stained with DAPI (blue). (**C**) The number of dots per cell are represented as a box-plot graph. The number of HPV16 RNA dots per cell was counted in 80 and 62 cells for CTL and eIF4A3-depleted cells, respectively. One-tailed Student’s *t* test; ***P<0.001. (**D**) E6 RNA probe (red) and E7 RNA probe (gray) were detected as punctate dots in SiHa cells transfected with the pEYFP-eIF4A3 vector for 24 h. Nuclei were stained with DAPI (blue). (**E**) The number of dots per cell are represented as a box-plot graph. The number of HPV16 RNA dots per cell was counted in 101 cells for CTL and 86 cells for GFP-eIF4A3. One-tailed Student’s *t* test; ***P<0.001.

To test whether eIF4A3 could affect the subcellular localization of HPV16 E6/E7 transcripts, we analyzed, using RNA-FISH, their subcellular localization in SiHa cells depleted for eIF4A3. We used two probe sets designed to recognize the E6 sequence (from nucleotides 2 to 476) and the E7 sequence (from nucleotides 563 to 846) (Figure 3A). In both conditions (siRNA-CTL and siRNA-eIF4A3), HPV16 E6 and E7 transcripts were detected as punctate dots in the cytoplasm, indicating that the eIF4A3 knockdown had no effect, neither on the nuclear export, nor on the subcellular localization of E6 or E7 mRNAs (Figure 3B). However, we observed a significant increase in punctate dots corresponding to E6 and E7 mRNAs when SiHa cells were depleted for eIF4A3. Indeed, approx. ten dots per cell were counted in eIF4A3-depleted cells compared with four dots per cell in control cells (Figure 3C). These findings indicated that eIF4A3 did not influence the localization of E6 and E7 mRNAs but regulated their cellular levels. To confirm these observations, a plasmid coding the eIF4A3 protein fused to an N-terminal EYFP protein was transiently transfected in SiHa cells and E6/E7 m*RNA levels were analyzed by RNA-FISH*. Interestingly, less cytoplasmic dots corresponding to E6 and E7 RNAs were detected in eIF4A3-YFP transfected cells compared with non-transfected cells (Figure 3D,E). Finally, to examine whether HPV16 transcripts were recruited to cytoplasmic RNA granules such as stress granules (SGs) or P-bodies, E6 RNA-FISH in SiHa cells transfected with either the peGFP-Dcp1 vector (Supplementary Figure S3) or the peGFP-MLN51 vector (Supplementary Figure S4) were performed. No colocalization between GFP-Dcp1 or GFP-MLN51 proteins and E6 RNAs were observed, indicating that E6 RNAs were not recruited in these RNA granules. Altogether, these data confirmed that eIF4A3 act as a negative regulator of HPV16 mRNA levels without altering their intracellular localization.

### The eIF4A3 protein associated with ribonucleoparticles containing HPV16 early transcripts

**Figure 4:**
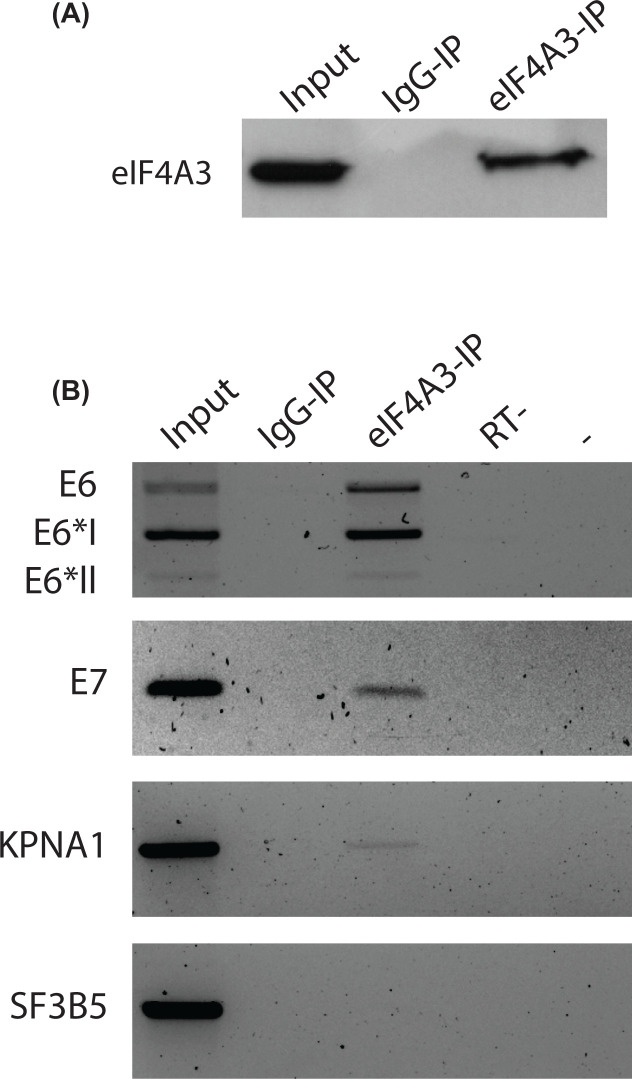
eIF4A3 associates with HPV16 E6/E7 transcripts (**A**) Western blotting analysis showing the immunoprecipitation of eIF4A3 protein in SiHa cells. (**B**) RNA ImmunoPrecipitation (RIP) analysis of RNAs from SiHa cells associated with either rabbit IgG control (IgG-IP) or eIF4A3 antibody (eIF4A3-IP). KPNA1 and the intronless SF3B5 mRNAs were used as positive and negative controls, respectively.

Next, we analyzed the interaction between the eIF4A3 protein and HPV16 mRNAs by performing RNA-IPs. Total RNA extracts from SiHa cells were incubated with anti-eIF4A3 (4A3-IP) or control (IgG-IP) antibodies, and the composition of the immunoprecipitated complexes were analyzed by Western blotting and RT-PCR (Figure 4A,B, **respectively**). As expected, the intronless SF3B5 mRNAs (negative control) were not immunoprecipitated, while the KPNA1 mRNAs (positive control) were detected to bind to eIF4A3 (Figure 4B). Taken together, these data validated our protocol of eIF4A3-targeted RNA-IP (4A3-IP). Interestingly, we observed a significant enrichment of E6/E7, E6*I/E7 and E6*II/E7 transcripts in the eIF4A3-IP compared with the control IgG-IP. Our results therefore confirmed that eIF4A3 proteins were indeed present in messenger ribonucleoprotein particles (mRNPs) containing HPV16 transcripts.

### NMD inhibitors, CHX and 5-azaC, differently affected HPV16 E6 and E7 mRNA and protein levels

**Figure 5:**
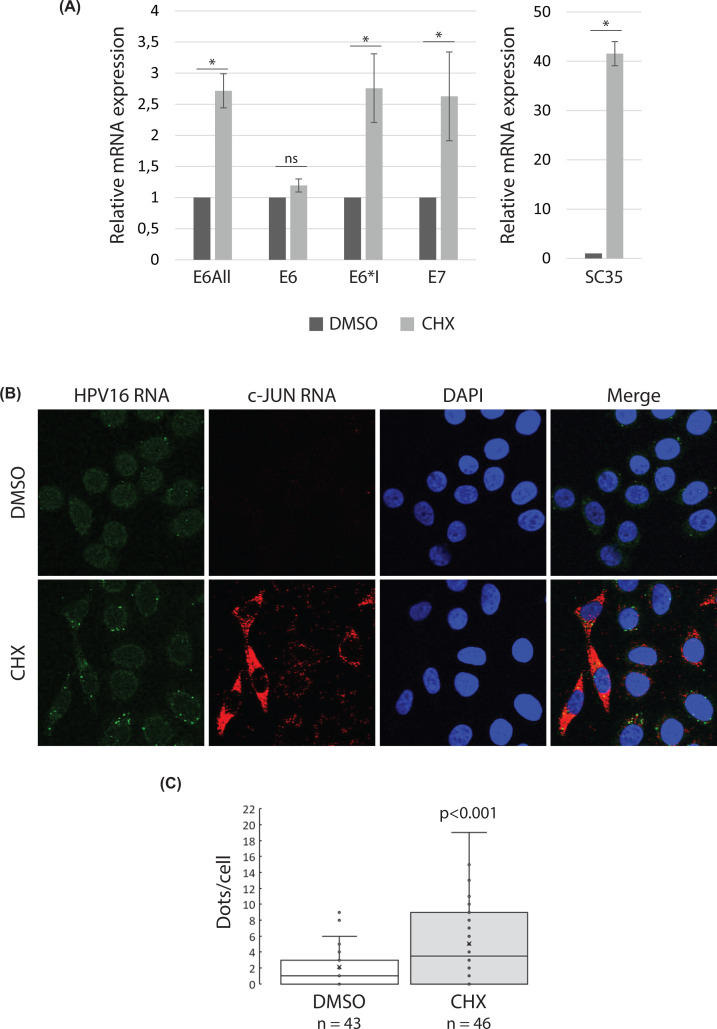
The inhibition of protein synthesis using CHX up-regulates HPV16 E6*I/E7 mRNA levels (**A**) RT-qPCR analysis of SC35 and HPV16 E6all, E6, E6*I and E7 mRNA levels in SiHa cells treated for 6 h with DMSO or CHX at 100 μg/ml. 18S was used for normalization. SC35 was used as a positive control of NMD inhibition. Data are represented as means of three independent experiments. Error bars = s.e.m.; *P<0.05; one-tailed Student’s *t* test; n.s. non-significant. (**B**) HPV16 (green) and c-JUN (red) transcripts were detected as punctate dots in DMSO- or CHX-treated SiHa cells using RNAscope and confocal microscopy. Data from one representative experiment of three independent experiments. (**C**) The number of HPV16 mRNA dots per cell was counted in 50 and 52 cells for DMSO and CHX treated cells, respectively, and are presented as a bar graph. p<0.001; One-tailed Student’s *t* test.

One possibility to explain how eIF4A3 depletion might lead to the up-regulation of HPV16 transcripts levels is its role in the NMD pathway. The NMD is responsible for the elimination of abnormal transcripts but also natural mRNAs which harbor NMD-eliciting features such as PTCs, multiple ORFs or long 3′-UTRs. The presence of multiple ORFs in HPV16 mRNAs and their multiple alternative splicing possibilities could therefore represent NMD-degradation signals. In order to determine whether the NMD pathway indeed regulated the levels of HPV16 transcripts, we explored the impact of drug-induced NMD inhibition. Because NMD requires translation, SiHa cells were first treated with CHX, a protein synthesis inhibitor, to inhibit NMD. As expected, the levels of SC35 mRNAs, a well-described physiological NMD substrate, were increased in CHX-treated cells compared with non-treated cells (Figure 5A). Interestingly, the CHX treatment had no effect on unspliced E6 RNA levels but E6*I and E7 RNA levels were up-regulated in CHX-treated cells compared with cells treated with DMSO alone (Figure 5A). Using *RNA FISH experiments* designed to detect HPV16 or c-JUN mRNAs (used as positive control) [48], we detected, as expected, an increase in the number of fluorescent signals corresponding to the positive control c-JUN mRNAs in CHX-treated SiHa cells compared with DMSO-treated cells. Regarding HPV16 mRNAs, we observed the presence of few cytoplasmic dots corresponding to HPV16 mRNAs (∼2–4 dots per cell) in DMSO-treated cells (Figure 5B) while, in CHX-treated cells, the number of dots corresponding to E6/E7 mRNAs was significantly increased (∼5–7 dots per cell) (Figure 5C).

**Figure 6:**
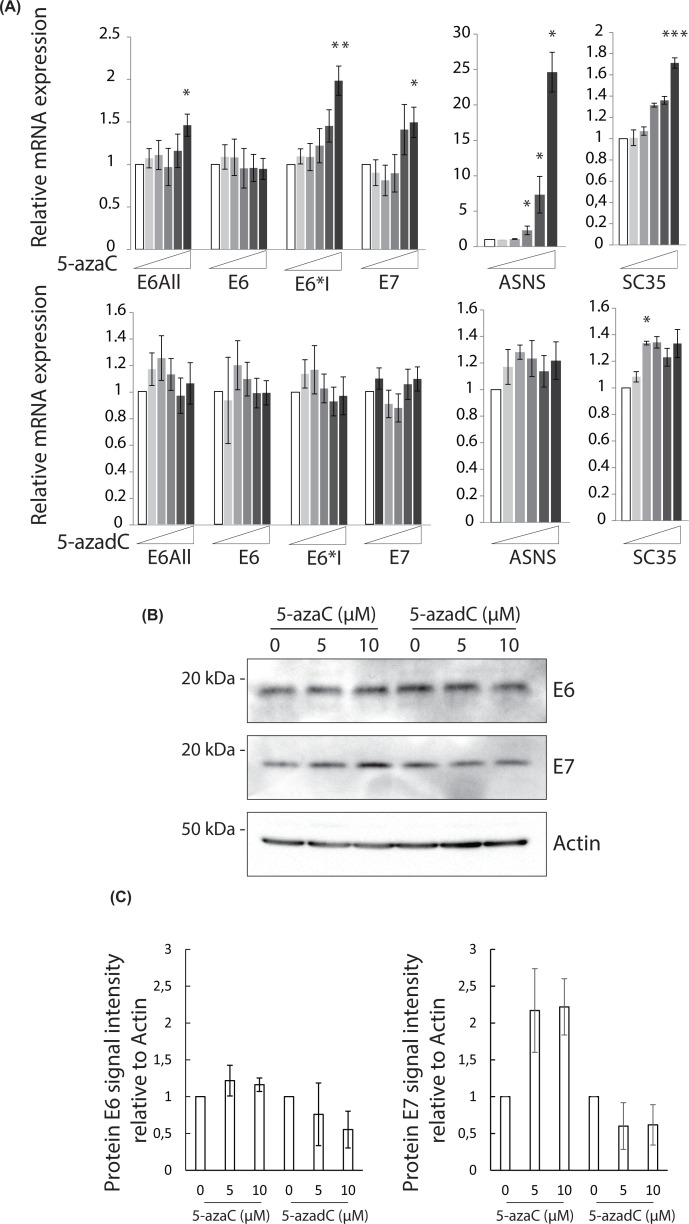
Up-regulation of HPV16 E7 expression in SiHa cells upon 5-azaC treatment (**A**) RT-qPCR analysis of ASNS, SC35 and HPV16 E6all, E6, E6*I and E7 expression in SiHa cells treated with increasing amount (0, 0.5, 1, 3, 5 and 10 μM) of 5-azaC (upper panel) or 5-azadC (lower panel) for 18 h. 18S was used for normalization. Data are represented as means of three independent experiments. Error bars = s.e.m.; *P<0.05; **P<0.01; ***P<0.001; one-tailed Student’s *t* test. (**B**) Western blotting analysis of E6 and E7 protein levels in SiHa cells treated with 0, 5 or 10 μM of 5-azaC or 5-azadC for 18 h. β*-*actin was used as a loading control. A representative blot is shown. (**C**) Relative quantification of E6 and E7 expression normalized with β*-*actin. The data are presented as mean values from two independent experiments. Error bars = s.e.m.

Recently, it has been reported that the inhibition of NMD in cells could also be obtained following a treatment using a nucleotide analog called 5-azaC [49]. Endogenous E6 and E7 mRNA and protein levels were thus monitored in SiHa cells treated with the active compound 5-azaC or the control compound 5-azadC, used as a negative control. Two physiological NMD substrates, the SC35 and asparagine synthetase (ASNS) mRNAs, were used as positive controls of NMD targets and we indeed showed an increase in their respective levels in the 5-azaC-treated cells (from a 1.4 to a 20-fold induction) (Figure 6A, **upper panel**) compared with the control 5-azadC-treated cells (Figure 6A, **lower panel**). No significant differences in the HPV16 mRNA levels were observed in cells treated with the 5-azadC compound (Figure 6A, **lower panel**). But, when cells were treated with 5-azaC, we detected a dose-dependent increase in E6*I and E7 mRNA levels while the E6 mRNA levels were not affected (Figure 6A **upper panel**). Moreover, we observed that the 5-azaC treatment significantly up-regulated the HPV16 E7 protein levels (two-fold change) while we did not detect any significant changes in the levels of the E6 protein (Figure 6B,C). As previously described, the demethylating agent 5azadC induces repression of E6 expression at both mRNA and protein levels [50]. Altogether, these data suggested that the alternatively spliced HPV16 transcripts encoding the E7 protein could be targeted by the NMD pathway to induce its degradation whereas the unspliced HPV16 transcript encoding the E6 protein might escape NMD-induced degradation.

## Discussion

The expression of the viral E6 and E7 oncoproteins is critical for the development and the maintenance of HPV-induced tumors. HPV16 transcripts generated from the p97 promoter allow for the production of the E6 and E7 proteins causing the inactivation of the tumor suppressor proteins *p53* and *pRb*, respectively. High E6 and E7 levels were linked to tumor growth and poor patient survival [51,52]. In contrast, low E6 and E7 levels restored antitumor mechanisms and induced tumor cell death [53,54]. Also, high levels of HPV16 spliced transcripts encoding the protein E6*I increased the levels of reactive oxygen species leading to tumor growth inhibition [55,56]. The expression of HPV16 transcripts is under the control of both viral and host cell factors. The main step of their regulation occurs at the post-transcriptional level through alternative splicing, leading to viral transcriptome and proteome diversification [16]. Nevertheless, HPV16 mRNA processing and the influence of RNA binding proteins in the control of viral RNA expression during infection and in the development of HPV-related cancer remain elusive. According to the TCGA database, we showed that the levels of the EJC factor eIF4A3 could be considered as an indicator of good prognosis in cervical cancers. Therefore, we addressed the role of this DEAD-box RNA helicase in the regulation of the expression of HPV16 early transcripts encoding E6 and E7 oncoproteins. For the first time, in the present study, we observed an up-regulation of HPV16 E7 mRNA and protein levels following eIF4A3 depletion and therefore, that eIF4A3 acted as a negative regulator of E7 expression.

Our data suggested that eIF4A3 might become an interesting target to regulate viral oncoprotein levels in HPV16-infected cancer cells. Previous studies showed that the inhibition of eIF4A3 could induce cell death by modifying the ratio between pro- and anti-apoptotic proteins. Indeed, the inhibition of eIF4A3 deregulated the splicing of several cellular transcripts including those encoding apoptosis-related factors, such as Bcl-X [25,33]. However, our current data indicated that the depletion of eIF4A3 did not alter the E6/E7 splicing pattern in the SiHa cell line leading us to the conclusion that the increased E7 levels, observed upon eIF4A3 depletion in the HPV16-positive SiHa cell line, were not a consequence of the alteration of their RNA splicing pattern.

During the course of RNA-FISH experiments, we detected more HPV16 transcripts in the cytoplasm of eIF4A3-depleted cells compared with the control ones. However, in our study, we did not observe any subcellular relocalization of HPV16 mRNAs in eIF4A3-depleted cells. In a previous publication, we observed that eIF4A3 accumulated in the nucleus, and more particularly in nuclear speckles, but, under stress conditions, eIF4A3 also partially relocalized in cytoplasmic SGs (Daguenet et al., 2012 and data not shown). SGs are cytoplasmic membrane-less aggregates of untranslated mRNPs implicated in cell survival under stress conditions. More recently, it has also been shown that the inhibition of eIF4A3 might affect the cellular stress response pathway by decreasing the formation of SGs [57]. In our data, we did not observe a colocalization between HPV16 transcripts and SGs or P-bodies. It may, therefore, be interesting to analyze the subcellular localization of HPV16 transcripts during stress conditions to determine whether these transcripts could be recruited, or not, into SGs.

Then, we confirmed the presence of eIF4A3 in HPV16 E6/E7 mRNPs highlighting a direct impact of this protein on viral mRNA biology. These results are in agreement with those of Martínez-Salazar et al., 2014, whom identified eIF4A3 and EJC factors as binding partners of the HPV16 transcripts [58]. Among the various EJC-related functions of eIF4A3, its involvement in the NMD pathway might explain the differences in HPV16 transcript levels observed following eIF4A3 depletion. HPV16 transcripts contain multiple ORFs, a feature that has been described to target mRNA for degradation by the NMD. Indeed, it has been suggested that SRSF2, a splicing factor, may regulate E6 and E7 oncoprotein expression by protecting HPV16 transcripts from NMD-induced mRNA degradation [59]. Moreover, several previous studies have already described viral mRNA degradation induced by the NMD [60–65]. To assess the impact of the NMD on HPV16 E6/E7 mRNA and protein levels, we used CHX and 5-azaC to inhibit the NMD pathway. We observed that a CHX, or a 5-azaC treatment, did increase E6*I/E7 RNA levels, but not E6 RNA levels. Interestingly, these results suggested that the spliced E6*I/E7 transcripts could be targeted by the NMD, whereas the unspliced E6/E7 mRNAs could escape NMD-targeted mRNA degradation. These differences could be explained by the distance separating the two ORFs. Indeed, the E6 and E7 ORFs are separated by 2 nucleotides whereas 153 nucleotides are present between the E6*I and E7 ORFs. It has been recently shown that translation re-initiation is one mechanism by which PTC-containing transcripts can escape NMD [66]. So, an efficient translation re-initiation process between the E6 and E7 ORFs could protect these transcripts from degradation. Other mechanisms have also been described to protect viral mRNA from NMD. This is for instance the case for the Rous Sarcoma Virus RNAs which possess a *cis*-acting element protecting them from NMD degradation [63]. Further studies will therefore be needed to unravel the mechanism by which eIF4A3 can modulate E6/E7 mRNA stability. Such work would also allow us to ameliorate our knowledge of HPV16 mRNA regulation but also the functions of EJC factors, and especially those of eIF4A3, in this regulation.

Altogether, we showed, for the first time in the present study, that eIF4A3 is a key regulator of HPV16 gene expression, likely through its implication in the NMD pathway. Given the good prognosis linked to high levels of eIF4A3 in cervical cancers, it would be interesting in the near future to quantify E6 and E7 expression levels in patients and determine whether we can find a correlation with the levels of expression of eIF4A3. If this correlation can be detected, we could then hypothesize that a loss of eIF4A3 function, along the evolution of HPV-induced carcinogenesis, could favor an overexpression of these oncoproteins and cervical cancer development.

**Figure 7:**
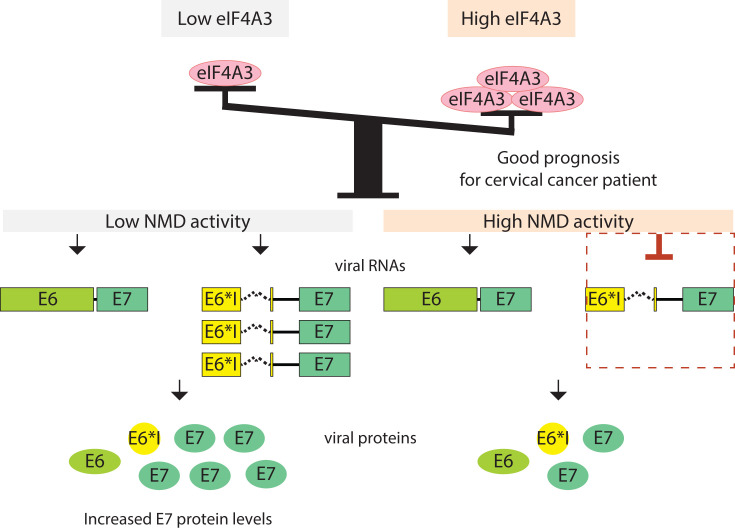
Model for the regulation of HPV16 transcripts by the eIF4A3 factor A decrease in NMD efficiency would increase E6*I/E7 mRNA levels leading to a high expression of the E7 protein. High eIF4A3 levels and high NMD activity would ensure low levels of E6*I/E7 mRNAs leading to a moderate E7 protein production. The colored boxes represent ORFs and color ovals represent the proteins generated from the corresponding transcripts.

According to our results, we can propose a model in which HPV16 transcripts can differentially elicit the NMD pathway. Indeed, high eIF4A3 levels and a high NMD activity could lead to low levels of E6*I/E7 mRNAs causing a moderate E7 protein production. On the other hand, low eIF4A3 levels and a decrease in NMD efficiency would lead to an increase in E6*I/E7 mRNA levels inducing a high expression of the E7 protein (Figure 7). Our data are in agreement with a recent study which showed that the HPV18 E7 protein can induce the expression of ABC transporters, such like P-gp, MRP1, MRP2 and BCRP, through a decrease in NMD efficiency in HPV-positive oropharyngeal cancer cells [67]. In addition, E6 and/or E7 protein have been described to affect NMD efficiency in order to favor E7 production and/or other viral proteins. Nevertheless, further studies will be required to determine whether the modulation of NMD efficiency during HPV infection could contribute to HPV-related carcinogenesis.

In conclusion, our data highlight the importance of RNA-binding proteins on HPV16 gene expression during the evolution of HPV-positive tumors and the question about their importance in the promotion of HPV-induced carcinogenesis.

## Supplementary Material

Supplementary Figures S1-S5 and Table S1

## Data Availability

The authors confirm that all data underlying the findings are fully available without restriction.

## Abbreviations

CHX: cycloheximide
DAPI: 4′,6-diamidino-2-phénylindole
eIF4A3: eukaryotic translation initiation factor 4A3
EJC: exon junction complex
EtOH: Ethanol
HPV: human papillomavirus
hrHPV: high-risk HPV
HRP: horseradish peroxidase
LCR: long control region
mRNP: messenger ribonucleoprotein particle
NMD: nonsense-mediated mRNA decay
ORF: open reading frame
PTC: premature termination codon
RT-qPCR: real-time reverse transcription polymerase chain reaction assays
SG: stress granule
TCGA: The Cancer Genome Atlas
5-azaC: 5-azacytidine
5-azadC: 5-aza-2′-deoxycytidine
