# Supplementary material for: The exon junction complex core factor eIF4A3 is a key regulator of HPV16 gene expression
Source: Biosci Rep. 2021 Apr 7;41(4):BSR20203488. doi: 10.1042/BSR20203488 (PMC8026852; doi:10.1042/BSR20203488)
Supplement: Supplementary Figures S1-S5 and Table S1 [file BSR-2020-3488_supp.pdf]

Supplementary Material

The Exon Junction Complex core factor eIF4A3 is a key regulator of HPV16 gene expression

Koceila Meznad[a,b,§], Philippe Paget-Bailly[a,b,d,§], Elise Jacquin[a,c], Anne Peigney[a,b,d], François Aubin[a,d,e], Michaël Guittaut[a,d,g], Christiane Mougin[a,b,f], Jean-Luc Prétet[a,b,e,f], Aurélie Baguet[a,d,g *]

[a] Université Bourgogne Franche Comté, France
[b] EA3181, UFR Santé, F-25000, Besançon, France.
[c] INSERM, U1231, Université Bourgogne Franche Comté, Dijon, France.
[d] INSERM, UMR1098, Interactions Hôte-Greffon-Tumeur/Ingénierie Cellulaire et Génique, F-25000, Besançon, France.
[e] Centre Hospitalier Régional Universitaire, 3 Bvd Alexandre Fleming, Besançon, France.
[f] Centre National de Référence Papillomavirus.
[g] DimaCell platform, Université Bourgogne Franche-Comté, F-25000 Besançon, France

[§] These authors contributed equally to this work.

[*]Corresponding author: Aurélie Baguet, INSERM, EFS BFC, UMR1098, Interactions Hôte-Greffon-Tumeur/Ingénierie Cellulaire et Génique, F-25000, Besançon, France.
Tel: +33 3 63 08 22 65
E-mail: aurelie.baguet@univ-fcomte.fr

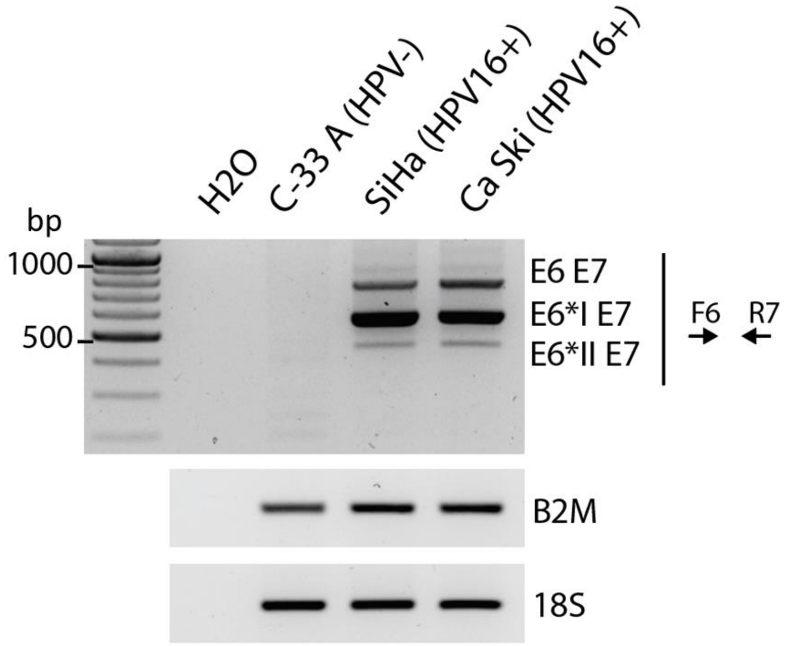

Figure S1: **Splicing pattern of HPV16 early transcripts.** RT-PCR showing alternative splicing patterns of HPV16 E6/E7 transcripts in SiHa and ca Ski cell lines. C-33A is an HPV-negative cell line derived from cervical carcinoma. B2M and 18S were used as loading controls. Agarose gel images are representative of two independent experiments.

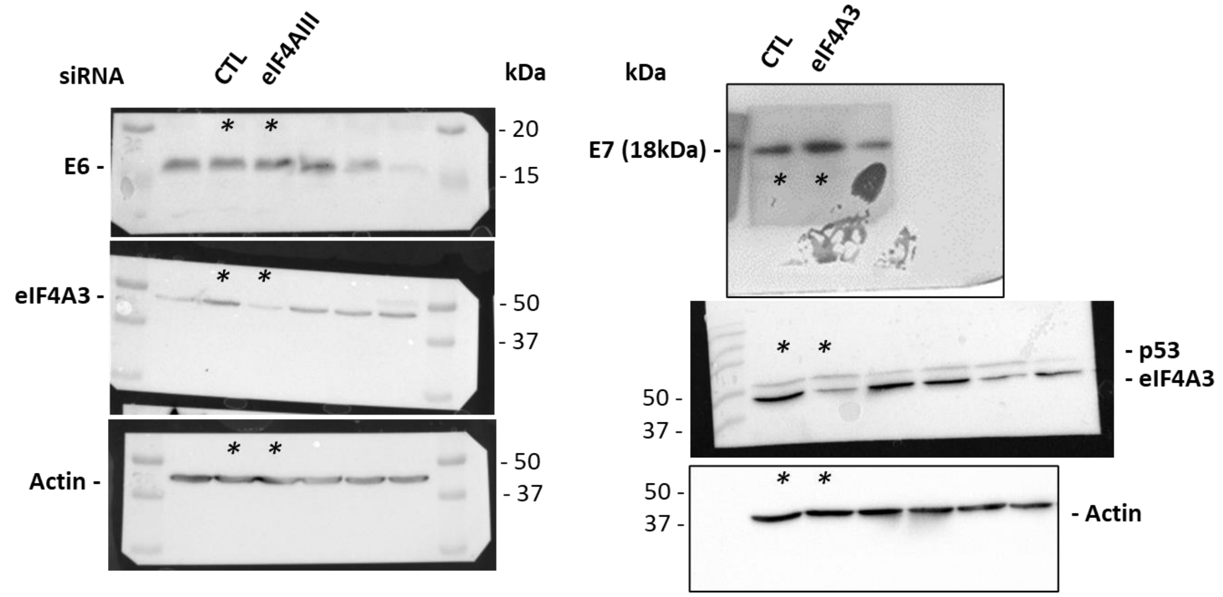

Figure S2: Uncropped Western blotting used in figure 2. Analyses showing endogenous E6, E7, eIF4A3 and β-actin protein levels in CTL or eIF4A3-depleted SiHa cells. * correspond to the lane displayed in figure 2.

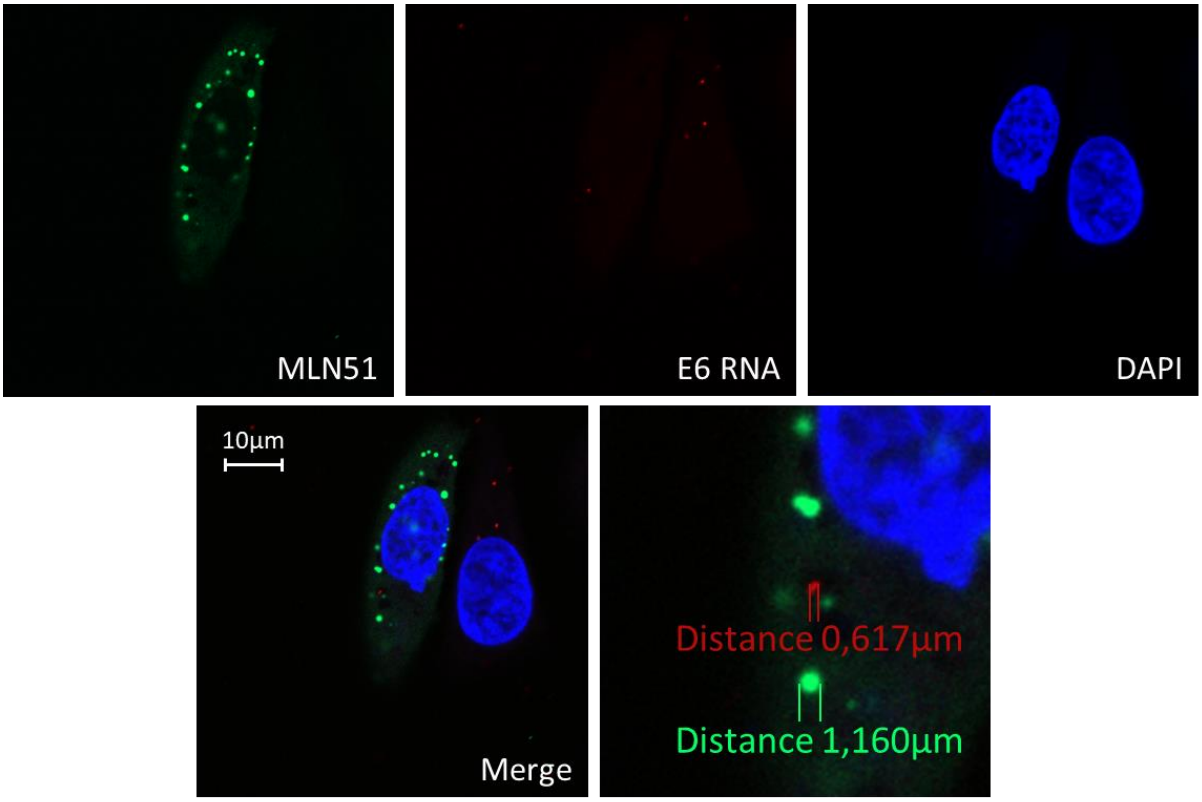

Figure S3: HPV16 E6 RNAs do not colocalize with stress granules. HPV16 E6 RNA-FISH were performed with SiHa cells transfected with the peGFP-MLN51 vector. MLN51 was recruited to stress granules. The image corresponds to a Z-section generated by confocal microscopy. Insets represent images enlargements and indicate the size of MLN51 and E6 RNA dots.

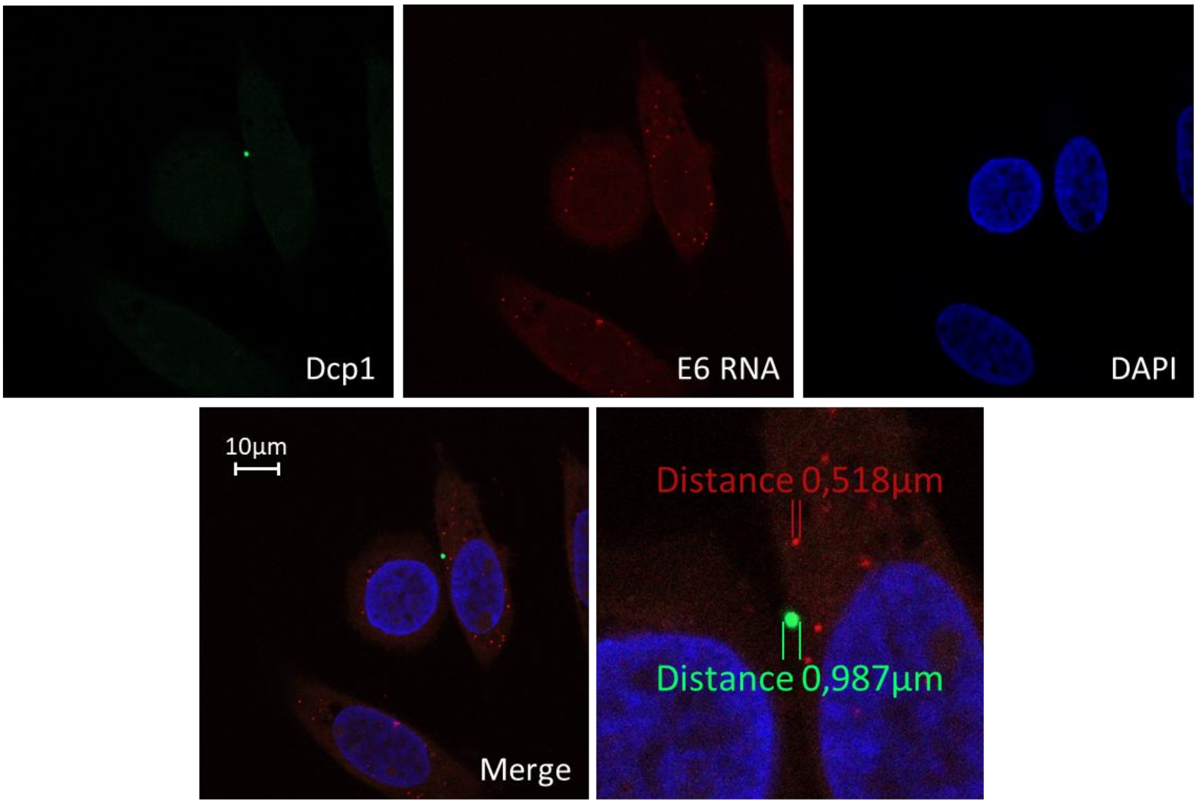

Figure S4: HPV16 E6 RNAs do not colocalize with P-bodies. HPV16 E6 RNA-FISH were performed with SiHa cells transfected with the peGFP-Dcp1 vector. Dcp1 was recruited to P-bodies. The image corresponds to a Z-section generated by confocal microscopy. Insets represent images enlargements and indicate the size of Dcp1 and E6 RNA dots.

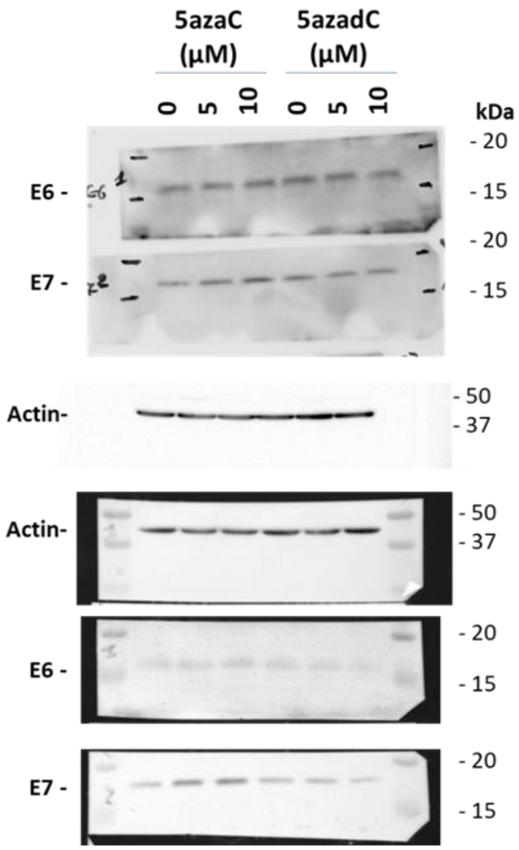

Figure S5: Uncropped Western blotting used in figure 6. Analyses showing endogenous E6, E7 and β-actin protein levels in SiHa cells treated or not with increasing dose of 5azaC or 5azadC. The upper blot is displayed in figure 6B and was revealed on an autoradiographic film. The lower blot is from a second independent experiment revealed with Chemidoc™ camera.

Table S1. PCR and qPCR primers used in the study.

Primer	Forward sequence (5'-3')	Reverse sequence (5'-3')	Use
16E6all	GCAATGTTTCAGGACCCACA	TTGTTTGCAGCTCTGTGCAT	qPCR
16E6	TGACTTTGCTTTTCGGGATTT	ACAGCATATGGATTCCCATCTC	qPCR
16E6*I	CTGCGACGTGAGGTGTATTA	TGTCCAGGTGTCTTTGCTTT	qPCR
16E7	ACAAGCAGAACCGGACAGAG	GCCCATTAACAGGTCTTCCA	qPCR and PCR
rRNA18S	GCAATTATTCCCCATGAACG	GGCCTCACTAAACCATCCAA	qPCR and PCR
eIF4A3	ACGAGCAATCAAGCAGATCA	AGGTGGCTGTTTTTCCTGTG	qPCR
SC35	CGGTGTCCTCTTAAGAAAATGATGTA	CTGCTACACAACTGCGCCTTTT	qPCR
ASNS	GGAAGACAGCCCCGATTTACT	AGCACGAACTGTTGTAATGTCA	qPCR
SF3B5	ACCGCTACACCATCCATAGC	AGGCTGAAGCATCTTTTCCA	PCR
B2M	GATGAGTATGCCTGCCGTGTG	CAATCCAAATGCGGCATCT	PCR
KPNA1	AGTGGTTTCTCCTGCTTTGC	GCTGATCCTCCAGAAGTTGC	PCR
E6E7ORF	ATGCACCAAAAGAGAACTGC (F6)	GCCCATTAACAGGTCTTCCA (R7)	PCR
